# Estimating micro area behavioural risk factor prevalence from large population-based surveys: a full Bayesian approach

**DOI:** 10.1186/s12889-016-3144-4

**Published:** 2016-06-07

**Authors:** L. Seliske, T. A. Norwood, J. R. McLaughlin, S. Wang, C. Palleschi, E. Holowaty

**Affiliations:** Analytics & Informatics, Cancer Care Ontario, 620 University Avenue, Toronto, ON M5G 2L7 Canada; Dalla Lana School of Public Health, Health Sciences Building, 155 College Street, 6th Floor, Toronto, ON M5T 3M7 Canada; Public Health Ontario, 480 University Avenue, Toronto, ON M5G 1V2 Canada; Lambton Public Health, 160 Exmouth Street, Point Edward, ON N7T 7ZT Canada

**Keywords:** Spatial epidemiology, Bayesian methods, Micro area analysis, Behavioural risk factors

## Abstract

**Background:**

An important public health goal is to decrease the prevalence of key behavioural risk factors, such as tobacco use and obesity. Survey information is often available at the regional level, but heterogeneity within large geographic regions cannot be assessed. Advanced spatial analysis techniques are demonstrated to produce sensible micro area estimates of behavioural risk factors that enable identification of areas with high prevalence.

**Methods:**

A spatial Bayesian hierarchical model was used to estimate the micro area prevalence of current smoking and excess bodyweight for the Erie-St. Clair region in southwestern Ontario. Estimates were mapped for male and female respondents of five cycles of the Canadian Community Health Survey (CCHS). The micro areas were 2006 Census Dissemination Areas, with an average population of 400–700 people. Two individual-level models were specified: one controlled for survey cycle and age group (model 1), and one controlled for survey cycle, age group and micro area median household income (model 2). Post-stratification was used to derive micro area behavioural risk factor estimates weighted to the population structure. SaTScan analyses were conducted on the granular, postal-code level CCHS data to corroborate findings of elevated prevalence.

**Results:**

Current smoking was elevated in two urban areas for both sexes (Sarnia and Windsor), and an additional small community (Chatham) for males only. Areas of excess bodyweight were prevalent in an urban core (Windsor) among males, but not females. Precision of the posterior post-stratified current smoking estimates was improved in model 2, as indicated by narrower credible intervals and a lower coefficient of variation. For excess bodyweight, both models had similar precision. Aggregation of the micro area estimates to CCHS design-based estimates validated the findings.

**Conclusions:**

This is among the first studies to apply a full Bayesian model to complex sample survey data to identify micro areas with variation in risk factor prevalence, accounting for spatial correlation and other covariates. Application of micro area analysis techniques helps define areas for public health planning, and may be informative to surveillance and research modeling of relevant chronic disease outcomes.

**Electronic supplementary material:**

The online version of this article (doi:10.1186/s12889-016-3144-4) contains supplementary material, which is available to authorized users.

## Background

A major goal of public health is to reduce the burden of preventable chronic diseases through healthy public policy, and programs and services designed and delivered at multiple societal levels (i.e., individual, inter-individual, community, regional, provincial and/or national). Two of the most common behavioural risk factors associated with this burden include excess bodyweight (overweight or obese) and tobacco use. The health risks of being overweight or obese include cardiovascular disease, stroke, type 2 diabetes, hypertension, osteoarthritis and several types of cancer [1]. Approximately 51 % of Ontario’s adults are overweight or obese, and over the past four decades, the prevalence has increased dramatically [2]. In spite of recent declines, the epidemic of tobacco use is far from being eradicated in Ontario [3]. Over 1.4 million Ontarians (12.6 %) still smoke, and it is estimated that approximately 13,000 tobacco-related premature deaths occur annually in Ontario, due to cancer, cardiovascular disease and respiratory disease [3, 4]. Notwithstanding the overall declines in the prevalence of tobacco use, it is likely that social inequalities in tobacco use will persist, perpetuating disparities in health.

The targeting of local health needs is a key principle for the delivery of public health services which, in Ontario, is mandated formally in the “Ontario Public Health Standards” [5]. The organizations that deliver front-line public health programs (Public Health Units - PHUs) are required to establish need through population health assessment and surveillance and to continuously tailor programs and services based on the needs identified. Obesity and tobacco control programs that address the specific needs of communities are likely to be more effective than broadly based programs planned and delivered at higher scales of geography, such as the county- or province/state-level [6, 7]. Built environments that promote physical inactivity (i.e., sedentary jobs, poor walkability, and the absence of green spaces) and the consumption of energy-dense foods (i.e., junk foods, larger portion sizes) are major determinants of obesity [1]. Likewise, built environments that promote the sale and use of tobacco products are major determinants of tobacco use [3]. The shortage of resources for local public health programs heightens the need for micro area data on risk factor prevalence in order to support the planning and evaluation of programs delivered to communities with the greatest need.

To date, surveillance of risk factors across the population has relied largely on the design and implementation of complex sample surveys, such as the Canadian Community Health Survey (CCHS) [8], Centers for Disease Control’s Behavioral Risk Factor Surveillance System (BRFSS) [9], the European Information Campaign on Diet and Nutrition (EURALIM) [10], New South Wales Population Health Survey [11], Italy’s Behavioral Risk Factor Surveillance System (STEPS) [12], the Finbalt Health Monitor [13], and Ontario’s Rapid Risk Factor Surveillance System (RRFSS) [14]. These surveys readily provide high quality data at the health region or PHU level, but not at the community level (i.e., village, town, or subdivisions of larger cities). Smaller areas typically do not have adequate sample sizes for directly calculating prevalence rates with reasonable precision, and the release of simple counts may pose a privacy risk. Accordingly, a standard practice in the publication and release of survey data is to specify whether a particular estimate’s coefficient of variation is indicative of “acceptable quality”, as compared with being marginal or unacceptable [8, 15].

Thus, a key challenge in using sample surveys to estimate risk factor prevalence for small geographic areas is from managing few observations, whether in the numerator (health events; sample survey responses) or denominator (population counts). Advanced analysis techniques, such as spatial smoothing, can address this challenge by including information from adjacent geographic areas to obtain stable estimates of rates or ratios [16–19] and can reveal heterogeneity in patterns of health that cannot be detected at larger spatial levels. Additionally, several spatial smoothing methods have the capacity to control for covariates, which may provide more valid measures of health by accounting for underlying variations in the distribution of important confounders such as age, income or other relevant factors.

The rationale for this study was to generate micro area covariate-adjusted estimates of behavioural risk factor prevalence estimates within the Erie-St. Clair Local Health Integration Network (LHIN) in order to provide information for targeted public health programs and to inform future micro area models of chronic disease. The LHIN covers a population of nearly 630,000 people in three contiguous PHUs located in southwestern Ontario, which align with the counties in this region. This estimation was accomplished through the use of a spatial Bayesian hierarchical model applied to sample survey data on height, weight and tobacco use, and other information from the CCHS and the Census of Canada [8, 20–24]. Key covariates such as an individual’s age and survey cycle and the micro area median household income were included in the modeling. Our objectives correspond to the general goals of disease mapping and spatial analysis [25, 26], which include displaying data and identifying patterns in their spatial distribution. For this study, a spatial Bayesian hierarchical model is applied with three main objectives:

1. To estimate the prevalence of obesity and current tobacco use at the micro area level across the Erie-St. Clair LHIN with acceptable precision and accuracy, while accounting for spatial correlation and potential confounders;
2. To identify areas of unusually high risk factor prevalence;
3. To describe the spatial distribution of these risk factor prevalence estimates (i.e., spatial trend and patterns of aggregation) over the entire region by sex;

## Methods

### Data sources

Data were linked from several sources to conduct this study. The sources included: i) behavioural risk factor data from the CCHS [8, 20–23], ii) micro area geographic boundary files [27], and iii) socio-demographic covariates for these small geographic areas [28].

### Canadian community health survey

The CCHS is a large cross-sectional survey of Canadians aged 12 years and over from all Provinces and Territories in Canada who live in private dwellings [8]. Approximately 98 % of the population is covered by the CCHS, which collects information on health status, health care utilization and determinants of health among Canadians [8, 20–23]. Excluded from the survey are those living on aboriginal reserves or Crown land, full-time members of the Canadian Armed Forces, as well as individuals living in institutions or in certain remote regions of Canada. Each sampling of the survey is named a “cycle”. The sampling methodology for the CCHS is complex and includes several steps. Households are selected for participation using two sampling frames: area-based sampling frames for health regions (in Ontario, health regions are the PHUs) and telephone-based sampling frames. Each person does not have an equal probability of being selected for the CCHS survey, therefore various factors are incorporated in constructing the CCHS sampling weights, including: the health region population, clustering of households, multiple telephone lines, the number of out-of-scope units, non-response (at both the individual- and household-level), and characteristics such as household size, age and sex [20–23]. The cycles of the CCHS used in the analysis were collected in 2000–2001, 2003, 2005, 2007–2008 and 2009–2010.

Survey questions relevant to current smoking and excess bodyweight were examined to ensure consistency across the various CCHS cycles prior to combining them. Respondents were classified as current smokers if they reported smoking cigarettes daily or occasionally at the time of the survey. Excess bodyweight status was determined from respondents’ self-reported heights and weights, to calculate body mass index (BMI; kg/m^2^) and was comprised of overweight or obese individuals. For adults, a BMI of 30 kg/m^2^ and above was used to classify individuals as obese, whereas a BMI between 25 and 30 kg/m^2^ was used to classify individuals as overweight [29]. Respondents were dichotomized based on having excess bodyweight (BMI ≥ 25 kg/m^2^) or not (BMI < 25 kg/m^2^), with exclusions given to pregnant and lactating women. For respondents 12 to 17 years of age, the International Obesity Taskforce thresholds were used [30], which are age- and sex-specific thresholds that correspond to the adult values of 25 and 30 kg/m^2^ for overweight and obesity, respectively.

The “share file” version of the CCHS provides the complete postal code for survey participants who agreed to share their data [8, 20–23], which enables linkage to other relevant datasets such as the Census of Canada for area-based analyses. Sampling weights for the share file are derived from the sampling weight in the master file containing all respondents, and are adjusted for the percentage of CCHS respondents (>90 %) who agree to share their data in the CCHS share file. However, sampling weights are available at the granularity of health regions only. An enhanced postal code conversion file (PCCF+) program by Statistics Canada assigned the postal codes to Census administrative units using a population-weighted random allocation method [31], described in detail elsewhere [16]. Although the CCHS is a large population-based survey, a single cycle may not have a sufficient number of respondents, particularly if the population of interest has a rare health characteristic or if a study involves small geographic areas [32]. Thus, five cycles of the CCHS from Cycle 1.1 (2000–2001) to Cycle 2009–2010 were combined using a pooled approach to increase precision.

Combining cycles increased the average number of respondents per unit area of analysis (described below). For example, the 2007–2008 CCHS cycle had 2,894 respondents from the Erie-St. Clair LHIN, which equates to less than 3 people per area, on average, whereas all five cycles together had 14,639 respondents, increasing the average number of people per area to 13.

### Geographic boundary files

2006 Census administrative units called Dissemination Areas (DAs) were selected as the micro area for this study. DAs are the smallest geographical unit for which Statistics Canada releases the complete set of Census data (i.e. age group, sex, income, immigration, education, etc.). They have populations of 400 to 700 people, on average [33], and thus correspond to population density in terms of geographic size. In urban areas, DAs correspond to city blocks and in rural areas they are defined by administrative or physical features and infrastructure such as rivers, roads and railways. Since the geographic size of the DAs is relatively small, the term micro area estimate is used to refer risk factor prevalence estimated at the DA level. Boundary files for the 2006 Census of Canada DAs were available from Statistics Canada’s website in Geographic Information Systems (GIS) format [27]. For the 2006 Census, the Erie-St. Clair LHIN study area was comprised of 1,111 DAs. However, one DA (Pelee Island) was excluded since it was an island and had no neighbours, which would result in inconsistent (i.e. non-smoothed) prevalence estimates compared to the other DAs.

### Area-level covariates

Low socioeconomic status (SES) has been associated with an increased prevalence of smoking, both at the individual [34, 35] and area-level [35, 36]. An inverse relationship between area-level SES and smoking prevalence is similar for the sexes [37]. For overweight and obesity, inverse relationships have been found for SES in women, but less evidence of a gradient has been noted in men [38, 39]. To account for the effects of SES, micro area median household income, obtained from the 2006 census [28], was included in the modeling. Income data were suppressed or unavailable for several micro areas within the study area and risk factor prevalence estimates were not obtained for these areas when income was included in the models.

### Analysis

#### Estimation of risk factor prevalence

Given the implementation of a spatial model, a crude preliminary assessment of spatial autocorrelation was conducted using a parametric bootstrapped Moran’s I, using unweighted observed and expected counts (based on the mean study area proportions) simulated from a negative binomial distribution using the DCluster package in the R statistical software program [40]. This implementation addresses concerns regarding the assumption of an asymptotic normal distribution which is often not satisfied [41]. Moran’s I provides an indicator of strength of clustering (or not), ranging in values from −1 to +1 for spatial dispersion vs. clustering, respectively, and a test of significance.

To estimate the micro area prevalence of behavioural risk factors, we employed the Besag, York and Mollié (BYM) model [42]. This hierarchical Bayesian model is widely used in disease mapping; it is robust and includes terms for spatially correlated and uncorrelated random effects for each geographical unit of analysis [16, 42–44]. The spatially correlated term is important as 1) data aggregated over small spatial units often exhibit spatial correlation [45], and 2) it pools information from adjacent areas, which adds stability to the micro area estimates [46].

Each risk factor was modeled at the individual-level as a binary outcome, and included various parameters to calculate covariate-adjusted estimates of the behavioural risk factor probability within each micro area, described in detail in Additional file 1. Briefly, the individual-level estimates were obtained using linear combinations specific to sex, cycle, age-group for model 1, and sex, cycle, age group and micro area median household income for model 2. Using a post-stratification process (Additional file 1), these estimates were weighted to the micro area population structure, in consideration of the complex sampling, and then aggregated to obtain micro area prevalence estimates [47]. We refer to these estimates as “model-based”, whereas the direct survey sampling estimates, estimable at the PHU level, are referred to as “design-based”.

Bayesian inference was conducted using the WinBUGS software program [48] in conjunction with the glmmBUGS package in the R statistical program [40]. The Markov Chain Monte Carlo (MCMC) sampling method was used with the Gibbs sampling algorithm [49]. Models were run with three chains, and the first 500,000 samples were discarded as burn-in. An additional 50,000 samples were run, with the 10th iteration saved from each chain. A total of 5,000 samples were saved from each of the three chains, for a grand total of 15,000 samples for each micro area. Diagnostic plots for the random effect parameters, coefficients and standard deviations of the model were produced in WinBUGS [48] to assess chain convergence. Traceplots were examined to ensure that the three chains mixed adequately for the saved samples. In addition, Gelman-Rubin and autocorrelation plots were used to assess chain convergence and the degree of correlation between samples, respectively. Lastly, estimate sensitivity to the choice of priors was tested using Gamma and uniform hyperpriors (Additional file 1).

The Deviance Information Criterion (DIC) [50], a measure of goodness-of-fit for Bayesian models, was used to compare fit between models 1 and 2. Although there is no established threshold for DIC changes, a difference greater than 7 generally suggests meaningful differences between models [51, 52], with lower values indicating better model fit.

#### Accuracy and precision of prevalence estimates

To validate the model-based results, the estimates were aggregated to the PHU and health region and compared to the design-based estimates. For the design-based approach, the five cycles of the CCHS were combined together to create a pooled sample following the methods outlined by Thomas and Wannell [32]. When combining survey cycles, the total pooled population is several times larger than the population of each cycle. To have a pooled population size proportional to each cycle, the survey weights in the CCHS share file were re-scaled based on their weighted population size for each cycle. For the variance estimation, the CCHS bootstrap weights provided by Statistics Canada were similarly re-scaled for each cycle in the pooled sample.

The coefficient of variation (CV), calculated as the standard error of the micro area prevalence estimate divided by its mean, was used to evaluate Bayesian model precision [53]. We used the Statistics Canada threshold values for CCHS design-based estimates: a CV of less than 16.6 % was considered acceptable, between 16.6 and 33.3 % was considered marginal, and a CV above 33.3 % was considered to have low or unacceptable precision [8, 20–23]. To visualize the precision of the model-based risk factor prevalence estimates, they were graphed with their 95 % credible intervals (CIs) for each micro area for models 1 and 2.

#### Identification of high risk factor prevalence

In the Bayesian approach, the results are referred to as posterior distributions. These distributions can be examined and credible intervals obtained, which are akin to confidence intervals in the frequentist statistical approach. This is an advantage of the Bayesian approach as comparisons of each estimate relative to a baseline risk or referent group provides a measure of its “significance” and precision [54]. These posterior distributions were used to identify micro areas where 95 % or more of the MCMC simulation-based risk factor prevalence estimates exceeded the modeled average for the Erie-St. Clair LHIN. We refer to these as posterior probabilities. Pearson’s correlation coefficients were calculated to assess the relationship between precision (CVs) and elevated risk factor prevalence estimates (posterior probabilities).

To corroborate findings from the Bayesian modeling, separate SaTScan analyses were conducted using the raw postal code level CCHS data classified as binary outcome, with respondents either exhibiting the presence or absence of the behavioural risk factor of interest. The analyses were implemented using the Bernoulli distribution. SaTScan utilizes a spatial scanning window to evaluate whether the number of observations with an outcome was higher than expected via a maximum likelihood estimation [55]. We used an elliptical scanning window, with the maximum spatial cluster size set to 10 % of the population at risk in consideration of the irregular shape of the study area (Additional file 2). A total of 999 Monte Carlo replications were conducted for each analysis.

#### Description of risk factor distribution and trends

The spatial patterns of interest are those micro areas identified with high risk factor prevalence in excess of the study area average prevalence (i.e. high posterior probabilities), presented on all maps. To determine whether the prevalence of the risk factors exhibited linear trend over time, the models were also fit using CCHS cycle as an integer variable, to test the null hypothesis that the coefficient was not significantly different from zero.

For all statistical tests, alpha was set to 0.05 to minimize the type 1 error rate.

## Results

### Description of study area and CCHS respondents

Additional file 2 shows the geographical location of the Erie-St. Clair LHIN. Demographic and behavioural characteristics of the CCHS respondents in the region’s three counties (PHUs) are presented in Table 1. Across the five CCHS cycles, the county-level design-based current smoking prevalence ranged from 19.4 to 30.6 % in males, and 16.1 to 25.8 % in females. Excess bodyweight was more common in males, ranging from 52.0 to 67.8 % versus 36.3 to 45.9 % in females. The Moran’s *I* statistic was positive and significant, indicating spatial autocorrelation of similar values for all risk factors except excess bodyweight among males (Table 1). Additional file 3 displays the micro area Census-derived 2005 median household income by quintile, mapped to the 2006 Census DA boundaries. Areas of lower household income occurred within Chatham, Sarnia and Windsor, but also in some rural areas of Lambton and Chatham-Kent counties. Essex County tended to have rural areas with a higher household income. Income data were not available for 16 micro areas within the study area (Additional file 3).

**Table 1:** Demographic and Behavioural Characteristics of the CCHS Respondents by County in the Erie-St. Clair Region

	Males			Females		
	Lambton Total = 1,909	Chatham-Kent Total = 1,787	Essex Total = 3,013	Lambton Total = 2,216	Chatham-Kent Total = 2,144	Essex Total = 3,570
Age group, *N* (%)						
12–19	248 (13.0)	251 (14.0)	455 (15.1)	249 (11.2)	251 (11.7)	390 (10.9)
20–29	206 (10.8)	209 (11.7)	366 (12.1)	230 (10.4)	266 (12.4)	441 (12.4)
30–39	242 (12.7)	278 (15.5)	541 (18.0)	282 (12.7)	289 (13.5)	612 (17.1)
40–49	279 (14.6)	258 (14.4)	497 (16.5)	292 (13.2)	296 (13.8)	469 (13.1)
50–59	303 (15.9)	317 (17.7)	413 (13.7)	355 (16.0)	326 (15.2)	530 (14.8)
60–69	330 (17.3)	246 (13.8)	356 (11.8)	359 (16.2)	278 (13.0)	467 (13.1)
70–79	223 (11.7)	162 (9.10)	265 (8.8)	267 (12.0)	279 (13.0)	409 (11.5)
80 and over	78 (4.1)	66 (3.70)	120 (4.0)	182 (8.2)	159 (7.4)	252 (7.1)
CCHS cycle^a^, *N* (%)						
2000–2001 (Cycle 1.1)	369 (19.3)	472 (26.4)	561 (18.6)	470 (21.1)	544 (25.4)	672 (18.8)
2003 (Cycle 2.1)	406 (21.3)	318 (17.8)	619 (20.5)	439 (19.8)	412 (19.2)	710 (19.9)
2005 (Cycle 3.1)	385 (20.1)	344 (19.3)	626 (20.8)	423 (19.1)	385 (18.0)	755 (21.1)
2007–2008 (Cycle 4.1)	362 (19.0)	332 (18.6)	609 (20.2)	451 (20.4)	405 (18.9)	735 (20.6)
2009–2010	387 (20.3)	321 (18.0)	598 (19.8)	433 (19.5)	398 (18.6)	698 (19.6)
Percentage of current smokers (95 % CI)^b^						
2000–2001 (Cycle 1.1)	30.0 (24.4–35.6)	29.8 (25.1–34.5)	27.9 (23.6–32.2)	25.1 (20.0–30.3)	25.5 (20.8–30.3)	25.8 (22.4–29.3)
2003 (Cycle 2.1)	29.7 (23.7–35.8)	28.6 (22.0–35.3)	19.9 (16.0–23.7)	19.2 (14.1–24.3)	24.3 (18.8–29.9)	22.0 (17.8–26.2)
2005 (Cycle 3.1)	26.9 (21.6–32.2)	30.6 (24.3–37.0)	26.1 (21.8–30.3)	22.9 (17.6–28.2)	20.3 (14.9–25.6)	22.0 (17.7–26.2)
2007–2008 (Cycle 4.1)	23.9 (17.5–30.2)	29.2 (23.3–35.1)	19.4 (15.4–23.4)	24.1 (19.2–28.9)	25.3 (19.7–30.8)	19.1 (14.9–23.2)
2009–2010	24.6 (18.8–30.4)	29.5 (22.6–36.5)	27.8 (22.3–33.4)	20.4 (14.0–26.7)	16.7 (12.1–21.4)	16.1 (11.6–20.6)
Percentage with excess bodyweight (95 % CI)^b^						
2000–2001 (Cycle 1.1)	54.8 (48.8–60.7)	56.7 (51.7–61.7)	56.6 (52.2–61.0)	40.5 (35.7–45.3)	41.0 (36.2–45.9)	40.9 (36.6–45.2)
2003 (Cycle 2.1)	52.0 (45.8–58.2)	59.4 (53.2–65.6)	62.6 (57.5–67.7)	36.3 (29.6–43.0)	40.0 (32.7–47.3)	39.0 (34.0–43.9)
2005 (Cycle 3.1)	56.7 (50.5–63.0)	57.8 (53.2–62.5)	62.1 (57.2–67.0)	39.4 (33.4–45.3)	38.5 (32.9–44.1)	43.2 (38.7–47.7)
2007–2008 (Cycle 4.1)	56.3 (50.0–62.6)	60.9 (55.5–66.4)	60.0 (55.6–64.3)	41.4 (36.4–46.5)	41.5 (36.0–47.1)	39.8 (34.9–44.7)
2009–2010	65.0 (59.1–70.8)	64.7 (58.2–71.2)	67.8 (63.6–72.0)	41.4 (35.8–46.9)	45.9 (39.7–52.0)	43.2 (38.1–48.4)
Micro area “raw” % estimate Moran’s I (*p*-value), entire study area^c^						
Current smoking	0.06 (0.001)			0.06 (0.003)		
Excess bodyweight	−0.01 (0.684)			0.04 (0.013)		

*Abbreviations*: *CCHS* Canadian Community Health Survey, *CI* confidence interval

^a^After 2007–2008, the CCHS retired the numbered cycles (Cycle 1.1, 2.1, 3.1, 4.1) and used the years of data collection to identify the cycle instead. Both the survey year and numbered cycle (where relevant) are provided

^b^County-level prevalence estimates are weighted using the CCHS share file weights

^c^Raw estimates taken as the proportion of respondents with an affirmative response for the behavioural risk factor aggregated to the micro area (Dissemination Area). One-tailed test obtained from parametric bootstrap simulations of the observed and expected responses using a negative binomial distribution. *Provided only as a preliminary assessment of spatial autocorrelation*

### Micro area risk factor prevalence: accuracy and precision

The Bayesian modeling results were not sensitive to the choice of priors (Additional file 1). Estimate validity is presented in Table 2, which contrasts the design- and model-based post-stratified risk factor prevalence estimates for the Erie-St. Clair LHIN and its counties. Credible intervals are provided for the Bayesian model-based estimates, whereas confidence intervals are provided for the design-based estimates. For current smoking, model-based estimates were within the 95 % confidence intervals of the design-based estimates except male results for Essex County and the Erie-St. Clair region. All model-based estimates for excess bodyweight were within the 95 % confidence intervals of the design-based estimates. DIC values were lower for models that included micro area income (model 2). For current smoking, the DIC values were 92.4 lower for male estimates and 108.6 lower for female estimates; for excess bodyweight, the values were 79.3 and 103.6 lower for the prevalence estimates for males and females, respectively (data not shown).

**Table 2:** Design- and Model-Based Prevalence Estimates for Behavioural Risk Factors in the Erie-St. Clair Region, by County

	Lambton		Chatham-Kent		Essex		Erie-St. Clair Region	
	Males % (95 % CI)	Females % (95 % CI)	Males % (95 % CI)	Females % (95 % CI)	Males % (95 % CI)	Females % (95 % CI)	Males % (95 % CI)	Females % (95 % CI)
Current smoking								
Design-based^a^	27.5 (25.2–29.9)	22.3 (20.2–24.4)	29.2 (26.7–31.6)	23.0 (21.0–25.1)	23.5 (21.8–25.2)	21.8 (20.1–23.4)	25.1 (23.8–26.4)	22.1 (20.9–23.3)
Model 1^b^	28.5 (27.8–29.2)	20.6 (19.9–21.3)	29.2 (28.7–29.7)	21.9 (21.4–22.4)	25.8 (25.4–26.2)	21.3 (21.0–21.7)	27.1 (26.7–27.4)	21.3 (21.0–21.6)
Model 2^b^	27.7 (26.8–28.6)	21.8 (20.8–22.8)	28.9 (28.6–29.2)	23.4 (23.1–23.7)	26.2 (26.0–26.4)	22.7 (22.5–22.9)	27.1 (26.9–27.2)	22.6 (22.5–22.8)
Excess bodyweight								
Design-based^a^	58.4 (56.0–60.9)	43.5 (41.0–45.9)	61.4 (59.3–63.6)	46.4 (43.9–48.9)	61.3 (59.2–63.4)	46.6 (44.5–48.6)	60.8 (59.3–62.3)	45.9 (44.4–47.4)
Model 1^b^	57.9 (57.6–58.2)	44.6 (44.3–45.0)	60.3 (60.0–60.6)	46.6 (46.2–47.0)	61.5 (61.3–61.8)	45.7 (45.5–46.0)	60.5 (56.8–64.1)	45.6 (45.5–45.8)
Model 2^b^	58.0 (57.8–58.2)	44.5 (44.3–44.7)	60.2 (60.0–60.4)	46.7 (46.5–47.0)	61.6 (61.4–61.7)	45.7 (45.5–45.8)	60.5 (60.4–60.6)	45.6 (45.5–45.7)

To obtain a design-based pooled prevalence estimate across all 5 CCHS cycles, the cycle-specific survey weights for each respondent were re-scaled based on the proportion that cycle contributed to the total weighted pooled population using Statistics Canada’s Bootvar program (1). The re-scaled weights were used to calculate the design-based prevalence estimates. The model-based prevalence estimates were aggregated to the micro area using post-stratification methods (see Additional file 1). The mean risk factor prevalence was calculated for each county, as well as the entire region (LHIN) for both models. Model 1 results are comparable to the design-based estimates, which do not adjust for income

*a *The interval of variation of the design-based prevalence estimate is expressed as a 95 % confidence interval (CI). *b*The interval of variation of the Bayesian model-based prevalence estimate is expressed as a 95 % credible interval (CI).

*Abbreviations*: *CCHS* Canadian Community Health Survey, *LHIN* Local Health Integration Network

(1) Statistics Canada. Bootvar: User Guide (Bootvar 3.1 – SAS Version). Ottawa. 2005

The CVs indicated that, of the current smoking prevalence estimates from model 1, 1.0 % (10 of 1,035) of the micro areas had low precision among males, and 2.0 % (21 of 1,057) had low precision among females. The majority of the micro area estimates had marginal precision: 90.1 % (933 of 1,035 micro areas) for males and 96.5 % (1,020 of 1,057 micro areas) for females. However, including micro area income (model 2) for current smoking greatly improved precision as no estimates were considered to have low precision. For these estimates, precision was acceptable for 89.1 % (919 of 1,032) of the micro areas for males and 62.1 % (654 of 1,053) of the micro areas for females, and the remaining estimates were marginal.

The micro area prevalence estimates for excess bodyweight were more precise than current smoking: no estimates had low precision for models 1 or 2, for either sex. For model 2, all 1,033 micro areas with available data for males had acceptable precision, and 99.4 % (1,044 of 1,050) of micro areas for females were acceptable.

### Risk factor prevalence estimates and identification of areas with high prevalence

**Fig. 1:**
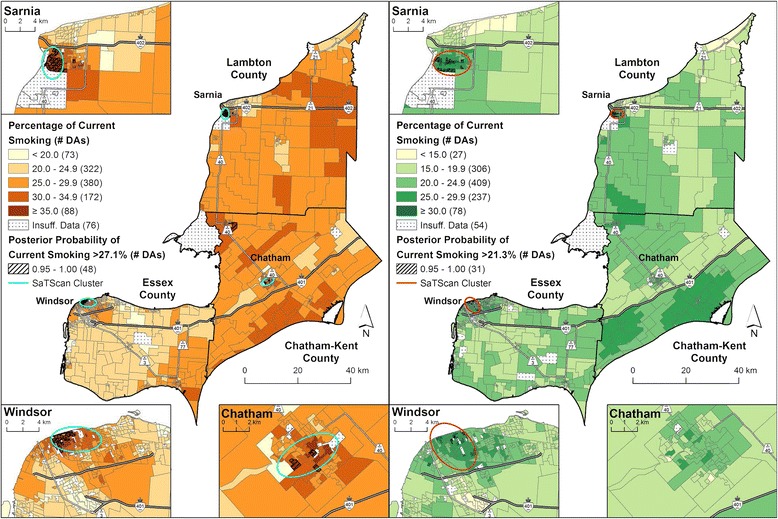
Current smoking prevalence by 2006 DA, adjusted for survey cycle and age (model 1). Males are shown on the *left* and females are shown on the *right*. Micro area prevalence of current smoking calculated as a percentage for 1,111 DAs using full Bayesian logistic regression with the Besag-York-Mollié (BYM) model to account for the spatially correlated and uncorrelated random effects. Posterior probabilities that exceeded the model-based Erie-St. Clair regional average for current smoking (males: 27.1 %, females: 21.3 %) for 95 % or more of the Bayesian simulations are shown with *hatch marks*. Statistically significant SaTScan clusters (*p*-value ≤ 0.05) are indicated by the *ellipses*

**Fig. 2:**
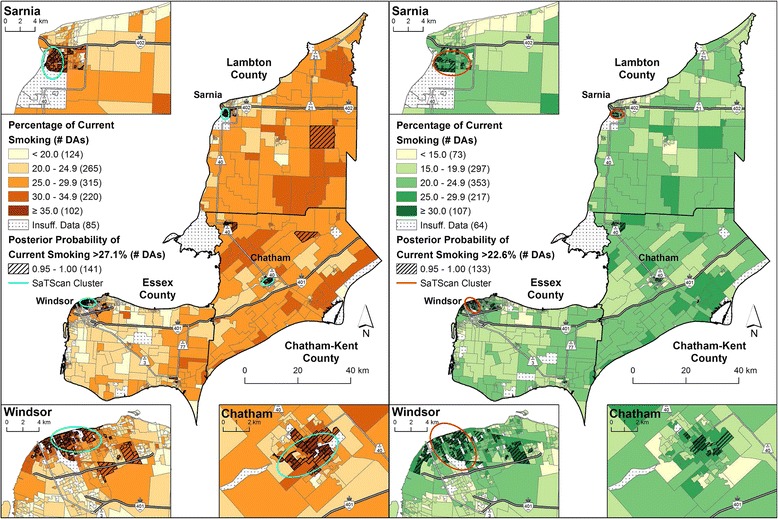
Current smoking prevalence by 2006 DA adjusted for survey cycle, age and household income (model 2). Males are shown on the *left* and females are shown on the *right*. Micro area prevalence of current smoking calculated as a percentage for 1,111 DAs using full Bayesian logistic regression with the Besag-York-Mollié (BYM) model to account for the spatially correlated and uncorrelated random effects. Posterior probabilities that exceeded the model-based Erie-St. Clair regional average for current smoking (males: 27.1 %, females: 22.6 %) for 95 % or more of the Bayesian simulations are shown with *hatch marks*. Statistically significant SaTScan clusters (*p*-value ≤ 0.05) are indicated by the *ellipses*

Figures 1 and 2 display the micro area current smoking prevalence estimates for models 1 and 2, respectively. Results for the male population from model 1 identified 48 micro areas in Sarnia, Chatham and Windsor with high posterior probabilities (i.e. the estimates exceeded 27.1 %, Table 2). For females, 31 micro areas in Sarnia and Windsor had high posterior probabilities (exceeded 21.3 %, Table 2). Results from model 2 showed that current smoking prevalence estimates for males were elevated in many micro areas within Sarnia, Chatham and Windsor, in addition to some rural areas, after adjusting for household income. A total of 141 micro areas had high posterior probabilities (exceeded 27.1 %, from model 2, Table 2). Results for current smoking prevalence estimates for females indicated a similar pattern, with 133 micro areas having high posterior probabilities (exceeded 22.6 %, Table 2).

SaTScan analyses corroborated the identification of these areas of elevated risk factor prevalence, detected in Sarnia, Windsor and Chatham for males, and Sarnia and Windsor for females (ellipses on Figs. 1 and 2, and Additional file 4). The Pearson correlation coefficient for the posterior probabilities and the CVs in were −0.49 and −0.45 for model 1 in males and females, respectively (*p*-value < 0.05 for both sexes). For model 2, the correlation coefficients were −0.49 for males and −0.65 for females (*p*-value ≤0.05 for both sexes).

**Fig. 3:**
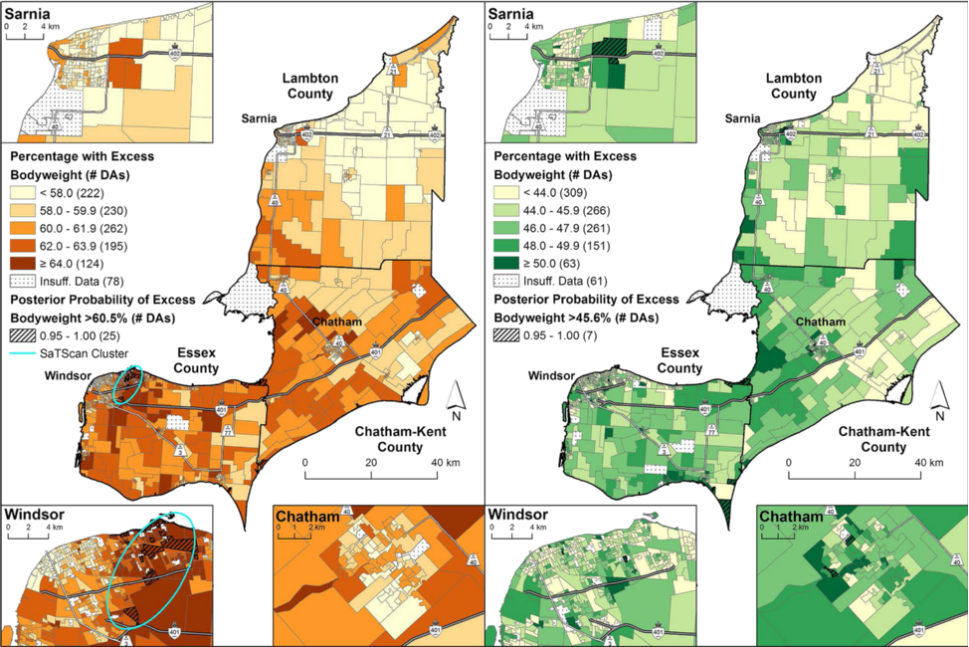
Excess bodyweight prevalence by 2006 DA, adjusted for survey cycle and age (model 1). Males are shown on the *left* and females are shown on the *right*. Micro area prevalence of excess bodyweight calculated as a percentage for 1,111 DAs using full Bayesian logistic regression with the Besag-York-Mollié (BYM) model to account for the spatially correlated and uncorrelated random effects. Posterior probabilities that exceeded the model-based Erie-St. Clair regional average for excess bodyweight (males: 60.5 %, females: 45.6 %) for 95 % or more of the Bayesian simulations are shown with *hatch marks*. A statistically significant SaTScan cluster (*p*-value ≤ 0.05) for males is indicated by the *ellipses*. No clustering was present for females

**Fig. 4:**
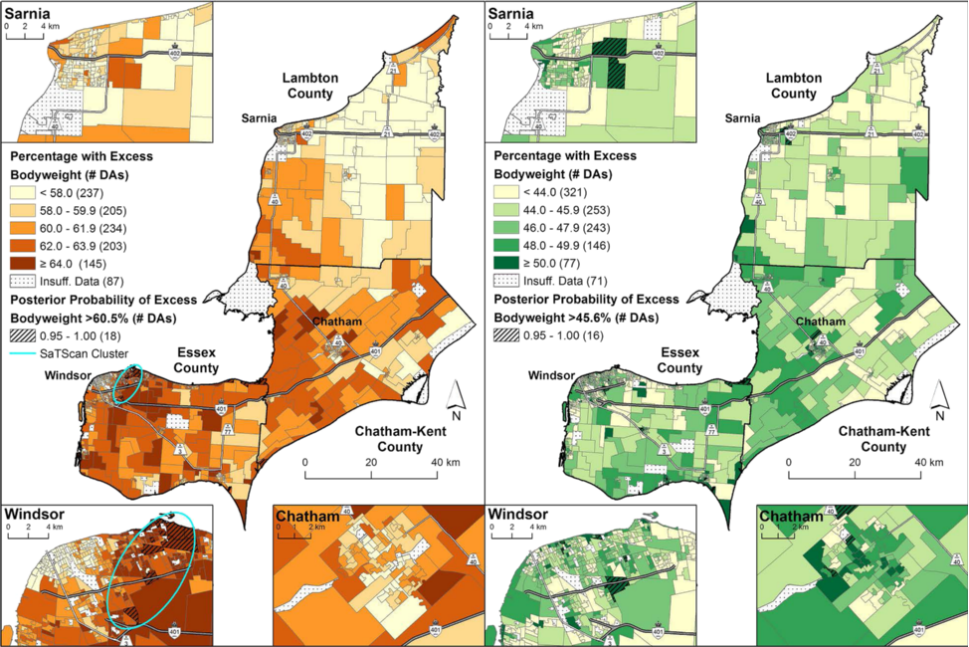
Excess bodyweight prevalence by 2006 DA, adjusted for survey cycle, age and household income (model 2). Males are shown on the *left* and females are shown on the *right*. Micro area prevalence of excess bodyweight calculated as a percentage for 1,111 DAs using full Bayesian logistic regression with the Besag-York-Mollié (BYM) model to account for the spatially correlated and uncorrelated random effects. Posterior probabilities that exceeded the model-based Erie-St. Clair regional average for excess bodyweight (males: 60.5 %, females: 45.6 %) for 95 % or more of the Bayesian simulations are shown with *hatch marks*. A statistically significant SaTScan cluster (*p*-value ≤ 0.05) for males is indicated by the *ellipses*. No clustering was present for females

Figures 3 and 4 show the post-stratified micro area prevalence estimates for excess bodyweight for models 1 and 2, respectively. Results for males from model 1 yielded 25 micro areas, primarily in Windsor, with a high posterior probability (exceeded 60.5 %, Table 2). Results for females identified 7 micro areas with high posterior probabilities (exceeded 45.6 %, Table 2). Fitting micro area income in model 2 for males resulted in 18 micro areas exceeding the model-based average (60.5 %, Table 2), many of which were located in the Windsor area. Model 2 results for females yielded 16 micro areas with high posterior probabilities (exceeded 45.6 %, Table 2).

SaTScan identified a cluster of elevated prevalence estimates for excess bodyweight among males in Windsor, but no clusters for females (Figs. 3 and 4, and Additional file 4). The Pearson correlation coefficient between the posterior probabilities and the CVs for model 1 were −0.57 for males and −0.59 for females, and for model 2 − 0.63 and −0.67, respectively. All correlations were statistically significant (*p*-value ≤0.05).

Additional file 5 displays the effect of fitting micro area income on the precision of the prevalence estimates for current smoking and excess bodyweight, respectively. For current smoking among males, inclusion of neighborhood-level income (model 2) reduced estimate variance (i.e. narrower credible intervals) yielding higher posterior probabilities. Results for females were similar, with narrower credible intervals shown for model 2. For excess bodyweight, there were no visually discernable differences in the credible interval widths comparing model 1 and 2 for either sex.

### Risk factor prevalence spatial distribution, trends and description

Adjustment for micro area income (model 2) had a notable impact on the spatial distribution of the risk factor prevalence estimates. For current smoking, the inclusion of micro area median household income resulted in higher prevalence estimates and posterior probabilities in urban areas as well as some rural areas in Chatham-Kent and Lambton counties among males (Fig. 1 vs. Fig. 2). For excess bodyweight, an overall north-to-south gradient of increasing prevalence was evident for both sexes.

Table 3 provides the modeled odds ratios (ORs) for the behavioural risk factors from the individual-level models, comprised of individual-level (age group, CCHS cycle) and micro area covariates (median household income). For current smoking estimates among males and females, all age groups except 30–39 years for males had ORs significantly different from the reference group (aged 50–59), whereas the younger ages (excluding teenagers) typically had increased odds – 26 % or higher odds – and ages 60 and above at least 30 % lower odds of current smoking, after adjusting for the other covariates. From model 2, a $10,000 increase in micro area median income was associated with an approximately 12–13 % decrease in the odds of current smoking, after adjusting for cycle and age.

**Table 3:** Modeled Relationships Between Covariates and Behavioural Risk Factors for the Erie-St. Clair Region

	Current smoking		Excess bodyweight	
	Males OR (95 % CI)	Females OR (95 % CI)	Males OR (95 % CI)	Females OR (95 % CI)
Model 1:				
CCHS cycle^a^				
2000/2001 (Cycle 1.1)	1.00 (ref)	1.00 (ref)	1.00 (ref)	1.00 (ref)
2003 (Cycle 2.1)	0.87 (0.73–1.04)	0.85 (0.72–1.01)	1.14 (0.97–1.34)	0.87 (0.75–1.01)
2005 (Cycle 3.1)	0.98 (0.82–1.17)	0.89 (0.74–1.05)	**1.18 (1.00–1.38)**	1.03 (0.88–1.19)
2007–2008 (Cycle 4.1)	**0.84 (0.70–1.00)**	**0.80 (0.67–0.95)**	**1.18 (1.00–1.39)**	1.00 (0.86–1.16)
2009–2010	0.88 (0.73–1.05)	**0.71 (0.59–0.85)**	**1.44 (1.21–1.71)**	**1.18 (1.01–1.37)**
p-trend	0.08	**<0.01**	**<0.01**	**<0.01**
Age (years)				
12–19	**0.34 (0.36–0.42)**	**0.60 (0.47–0.75)**	**0.14 (0.11–0.17)**	**0.15 (0.12–0.18)**
20–29	**1.29 (1.05–1.56)**	**1.69 (1.38–2.04)**	**0.37 (0.30–0.45)**	**0.37 (0.31–0.45)**
30–39	1.16 (0.96–1.40)	**1.26 (1.04–1.52)**	**0.81 (0.67–0.98)**	**0.52 (0.44–0.62)**
40–49	**1.44 (1.19–1.72)**	**1.61 (1.33–1.94)**	**0.75 (0.62–0.90)**	**0.63 (0.53–0.74)**
50–59	1.00 (ref)	1.00 (ref)	1.00 (ref)	1.00 (ref)
60–69	**0.60 (0.49–0.74)**	**0.70 (0.57–0.86)**	0.97 (0.79–1.18)	1.02 (0.86–1.21)
70–79	**0.24 (0.17–0.32)**	**0.39 (0.30–0.49)**	**0.77 (0.61–0.95)**	0.91 (0.76–1.08)
80 plus	**0.10 (0.05–0.17)**	**0.17 (0.11–0.25)**	**0.45 (0.33–0.60)**	**0.65 (0.53–0.80)**
Model 2:				
CCHS cycle^a^				
2000/2001 (Cycle 1.1)	1.00 (ref)	1.00 (ref)	1.00 (ref)	1.00 (ref)
2003 (Cycle 2.1)	0.88 (0.74–1.05)	**0.83 (0.69–0.99)**	1.14 (0.97–1.34)	0.86 (0.74–1.00)
2005 (Cycle 3.1)	1.02 (0.85–1.20)	0.87 (0.73–1.03)	1.18 (1.00–1.38)	1.02 (0.88–1.19)
2007–2008 (Cycle 4.1)	0.88 (0.73–1.04)	**0.80 (0.69–0.95)**	1.17 (0.99–1.38)	1.00 (0.86–1.16)
2009–2010	0.89 (0.74–1.07)	**0.70 (0.58–0.83)**	**1.45 (1.22–1.71)**	**1.18 (1.01–1.38)**
p-trend	0.10	**<0.01**	**<0.01**	**<0.01**
Age (years)				
12–19	**0.34 (0.27–0.43)**	**0.61 (0.48–0.76)**	**0.13 (0.11–0.16)**	**0.15 (0.12–0.18)**
20–29	**1.27 (1.03–1.54)**	**1.64 (1.34–1.99)**	**0.37 (0.30–0.44)**	**0.37 (0.30–0.44)**
30–39	1.17 (0.97–1.40)	**1.26 (1.04–1.53)**	**0.79 (0.65–0.96)**	**0.52 (0.44–0.62)**
40–49	**1.44 (1.19–1.72)**	**1.62 (1.33–1.96)**	**0.74 (0.60–0.89)**	**0.63 (0.52–0.74)**
50–59	1.00 (ref)	1.00 (ref)	1.00 (ref)	1.00 (ref)
60–69	**0.61 (0.49–0.74)**	**0.68 (0.55–0.84)**	0.95 (0.77–1.17)	1.01 (0.85–1.20)
70–79	**0.24 (0.17–0.32)**	**0.37 (0.28–0.47)**	**0.76 (0.61–0.95)**	0.90 (0.75–1.07)
80 plus	**0.09 (0.04–0.16)**	**0.16 (0.11–0.24)**	**0.44 (0.33–0.59)**	**0.64 (0.52–0.79)**
Household income				
Per $10,000 increase	**0.88 (0.85–0.91)**	**0.87 (0.85–0.90)**	1.02 (0.99–1.04)	**0.97 (0.94–0.99)**

*Abbreviations*: *CCHS* Canadian Community Health Survey, *OR* odds ratio, *CI* credible interval, *ref* referent group

^a^After 2007–2008 the CCHS retired the numbered cycles (Cycle 1.1, 2.1, 3.1, 4.1) and used the years of data collection to identify the cycle instead. Both the survey year and numbered cycle (where relevant) are provided. Bolded values indicate that the 95 % credible interval excludes the null association (OR = 1.00)

For excess bodyweight results from model 1, recent CCHS cycles were associated with increased odds of overweight or obesity, more so for male respondents, after controlling for age group. Generally, age was an important explanatory covariate after adjusting for cycle, but with less consistency than for the current smoking results. Nonetheless, most age groups had a decreased odds of excess bodyweight compared to ages 50–59, ranging from approximately 85 % lower odds for teenagers, to no significant difference for ages 60–69, regardless of sex. From model 2 for females, increased micro area income was associated with 3 % lower odds of excess bodyweight, after adjusting for the other covariates. The addition of income in model 2 resulted in negligible changes in ORs for age and cycle for both behavioural risk factors, but it did increase credible intervals for the excess bodyweight results, yielding borderline non-significant results.

Finally, model results for CCHS cycle fit as an integer variable indicated a statistically significant decrease over time for female current smoking for both models. Excess bodyweight results implied a statistically significant increase over time for both sexes and models 1 and 2 (Table 3).

## Discussion

The main objectives of this study were to demonstrate the feasibility of pooling large and complex population-based surveys to estimate the micro area prevalence of current smoking and excess bodyweight, and to identify areas with significantly elevated risk factor prevalence. The estimates provided by the models were stable, as indicated by the convergence of the models and the lack of sensitivity to the priors. Furthermore, the consistency of the design- and model-based estimates suggest that the modeled estimates were a valid method of obtaining behavioural risk factor prevalence. When comparing the results between models 1 and 2, model 2 provided better estimates of risk factor prevalence for current smoking, as evidenced by the lower CVs and the narrower credible intervals (increased precision) and lower DIC values (better model fit). For excess bodyweight, the CVs and credible intervals were similar between models 1 and 2, but the DIC values suggested that model 2 provided a better model fit. Therefore, the results suggest the addition of other important covariates, such as income, may provide more precise risk factor prevalence estimates. These results, controlling for additional covariates that could not be obtained for design-based estimates, have an important role in estimating and understanding the distribution of current smoking and excess bodyweight prevalence for intervention activities. For example, additional micro areas in Windsor and Chatham were identified as having elevated current smoking prevalence for both sexes after adjusting for income (Fig. 1 vs Fig. 2).

To date, this study among the first to provide micro area risk factor estimates, particularly in Canada. Meng et al. [56], provided smoking prevalence estimates at the municipal level, but results from our study showed the spatial distribution of sex-specific current smoking and excess bodyweight prevalence at a very granular level within Erie-St Clair. This information may help inform localized public health activities, which evidence suggests is more effective that larger regional programs [6, 7]. Micro area spatial variation in risk factor estimates may be detected using global statistics of spatial autocorrelation. This correlation can lead to violations of assumptions in other statistical approaches. We do not promote the use of such statistics with unweighted data from surveys with sampling frames – the purpose was simply to demonstrate the appropriateness of a model that accounts for spatial dependence. Large variation in the prevalence estimates were evident (15 % range for current smoking and 6 % range for excess bodyweight), and areas of elevated prevalence were detected. In contrast, PHU-level estimates typically reveal only small variations in the prevalence of smoking and excess bodyweight. For example, in 2009–2010, the survey-based current smoking estimates had a range of only 1.8 % and the combined overweight and obesity rates had a range of only 1.1 % across the three counties that comprise the Erie-St. Clair region [57].

The region contains a diverse mixture of urban centers, smaller towns and rural communities and it is expected that greater variation exists at smaller levels of geography. The results of this study illustrate the total range in the prevalence of key risk factors within the LHIN, and the importance of adjusting for key covariates such as micro area income. Examining the patterns that arise when incorporating the covariates together will contribute to our understanding of how geographic disparities in risk factor prevalence are related to known socioeconomic disparities and provide evidence for targeted interventions and enhance our knowledge of priority populations in the region.

The full Bayesian model used in this study has some key advantages over obtaining risk factor prevalence directly from survey data (i.e. design-based estimates). The modeled results may identify detailed, high-resolution heterogeneity in risk factor prevalence that cannot be obtained when calculating rates for small geographical areas with small counts. Also, the model allows for covariates to be accounted for in the analysis, which may help explain whether differences in risk factor prevalence estimates may be due to underlying differences in socioeconomic status, for example. Finally, the posterior probabilities identified areas where there was strong statistical evidence that the prevalence estimates were higher than the regional average. Previous work has indicated that posterior probabilities of 70 to 80 % provide adequate coverage to identify elevated values [58]; therefore, our selection of 95 % and above is conservative and we are confident that we have identified areas of true excess risk factor prevalence, adjusting for key covariates. The identification of areas with a high risk factor prevalence was also supported by SaTScan, which typically located high prevalence estimates consistent with the high posterior probabilities.

The correlation coefficient results for each of the sex-specific models, which were inverse and fairly strong, indicate that, as expected, micro areas with lower CVs (i.e. higher precision) had higher posterior probabilities, and vice-versa. Therefore, for knowledge translation and exchange, results geared towards public health actions might categorize the model-based prevalence estimates to focus only on those micro areas with high posterior probabilities and categorize all other prevalence estimates as similar to the study area.

Currently, only one other study in Canada has used the CCHS to derive the prevalence of health behaviours, although for a larger geographical unit [56]. Thus, there is a need for future work to confirm the results of these studies and to explore the relationship between a range of behavioural risk factors and outcomes such as cancer, heart disease, asthma and other adverse health conditions at a granular level of geography.

There are some important limitations to our study that warrant consideration. One inherent limitation is that these survey data were self-reported, and evidence suggests people under-report smoking [59, 60] and obesity [61]. In addition, while combining cycles of the CCHS increases statistical power, the pooled population is artificial. We assume that this pooled population represents the underlying population. The CCHS cycles were not specifically designed to be pooled, and there may be changes over time in the underlying population which are not captured when pooling the data. We attempted to account for temporal changes by including the CCHS cycle as a covariate, but there may be other changes that are not captured in our models.

Another important limitation is the complex survey design, whereby individuals have an unequal probability of being sampled. Statistics Canada provides sample weights by health region to obtain representative prevalence estimates, but these are unavailable for small geographical units such as DAs (micro areas). We note that our study area corresponds with one of the health regions (Erie St. Clair), a sampling frame, and we have attempted to address this limitation by performing post-stratification of the modeled estimates to the micro area Census populations. An alternate approach would be to re-scale the survey weights within the health region (i.e. a sampling frame). However, WinBUGS software currently does not support sampling weights for Bayesian models, as noted elsewhere [56]. Inclusion of these weights may have helped ensure that the survey respondents were representative of the Erie-St. Clair health region.

Finally, the CCHS data were collected between 2000–2001 and 2009–2010, but the micro area income data was from the 2006 Census. Therefore, the assumption was made that income levels are relatively stable across time. Perhaps of more importance is that the income levels relative to each area are assumed to be stable, such that a high income area does not rapidly shift towards being a lower income area, or vice versa. The overall percentage of census tracts that were low-income remained stable over a 20-year period in Canada’s metropolitan areas (population >100,000 people), although a sub-analysis of the three largest cities (Toronto, Montreal and Vancouver) found some variation in the geographic stability of low-income census tracts over time [62]. However, little is known about the stability of income in micro areas in smaller cities and rural areas in Canada. Lastly, the micro area household income values, by definition, were not sex-specific. Future work to explore individual and area-based income estimates may yield additional insights and further increase estimate precision.

## Conclusions

Using a full Bayesian hierarchical model, we identified spatial patterns of behavioural risk factors for small geographical areas within the Erie-St. Clair LHIN, which accounted for spatial correlation and potential confounders. These estimates have potential to aid in the targeting of public health programs and interventions. This study is among the first to demonstrate the feasibility of micro area prevalence estimates based on sample survey data using a spatial model. The application of granular Bayesian spatial models is likely to be facilitated by the introduction of new methods, such as the integrated nested Laplace approximation (INLA) [63]. INLA provides rapid results compared to the MCMC methods utilized in this study.

Micro area spatial analyses have important implications for targeted public health planning as well as evidence generation for relationships between behavioural risk factors and relevant disease outcomes. With the global spread in risk factor surveillance and recent international efforts to share and standardize methods of data collection and analysis [64, 65], these new micro area methods of analysis should be closely examined and tested by public health agencies wishing to maximize the use of these surveys.

## Abbreviations

BMI, body mass index; BYM, Besag, York, Mollié; CCHS, Canadian community health survey; CI, credible interval or confidence interval, as specified; CV, coefficient of variation; DA, dissemination area; DIC, deviance information criterion; GIS, geographic information systems; INLA, integrated nested Laplace approximation; LHIN, local health integration network; MCMC, Markov Chain Monte Carlo; OR, odds ratio; PCCF, postal code conversion file; PHU, public health unit; SES, socioeconomic status.

## Additional files

Additional file 1: Model Structure. (PDF 302 kb) Additional file 2: Erie-St. Clair Region (PDF 192 kb) Additional file 3: Median Household Income from the 2006 Census for the Erie St. Clair LHIN, by 2006 Dissemination Area(PDF 1788 kb) Additional file 4: Spatial Clustering of Behavioural Risk Factors for CCHS Respondents in the Erie-St. Clair Region (PDF 30 kb) Additional file 5: Mean Micro Level Behavioural Risk Factor Prevalence Estimates and 95 % Credible Intervals for the Erie-St. Clair Region (PDF 143 kb)
