# Supplementary material for: Estimating micro area behavioural risk factor prevalence from large population-based surveys: a full Bayesian approach
Source: BMC Public Health. 2016 Jun 7;16:478. doi: 10.1186/s12889-016-3144-4 (PMC4897930; doi:10.1186/s12889-016-3144-4)
Supplement: Additional file 1: — Model Structure. (PDF 302 kb) [file 12889_2016_3144_MOESM1_ESM.pdf]

Additional File 1

Behavioural risk factor prevalence was estimated using the Besag, York and Mollié (BYM) model [1] to account for the spatial correlation in risk factor prevalence between adjacent areas. For this study, individual (i.e. "unit-level") survey responses were the level of modeling, but micro area prevalence estimates of current smoking and excess bodyweight were of interest for mapping and analysis. Unit-level data were responses to the Canadian Community Health Survey (CCHS) and micro area data were from the 2006 Census Dissemination Areas (DAs) [2]. The equations used to model prevalence estimates are detailed in this section. Modeling was done separately for males and females.

Model Specification

An individual's response to a dichotomized risk factor question from the CCHS survey was assumed to be associated with individual and micro area level factors. The following equations define the multi-level prevalence model in a full Bayesian approach. The first level of the Bayesian model is shown in equation 1:

$$y_{ijk} \sim \text{Bernoulli}\ (p_{ijk}) \quad (1)$$

Here, y_{ijk} is the self-reported individual response to a relevant risk factor question as a binary outcome variable (1=yes; 0=no) and p_{ijk} is the probability of an individual having the risk factor based upon an individual's age group *i* (*i*=1-8) and survey cycle *j* (*j*=1-5), and residence in micro area *k* (*k*=1–1,111). The number of micro areas (*k*) varied by sex and by risk factor depending on data suppression. For example, 1,026 micro areas had complete covariate data for the male current smoking model that included income (model 2).

In the next level, the logit of the binary outcome is predicted by individual and micro area level covariates in the form of the BYM model:

$$\log\left(\frac{p_{ijk}}{1-p_{ijk}}\right) = logit(p_{ijk}) \ = \alpha + \beta_i + \gamma_j + \delta x_k + \ u_k + \ v_k \tag{2}$$

where, p_{ijk} is the probability of the binary outcome for an individual of age group i, survey cycle j and residing in micro area k; the natural log is the link function; α is the individual-level intercept and includes the referent groups; β_i is a vector of coefficients for age group i; γ_j is a vector of coefficients for survey cycle j; δ is the coefficient for micro area income in vector x_k; and, u_k and v_k are the micro area level random effect terms. The BYM model random effects (u_k and v_k, specified below) account for micro area level variation in the outcomes not explained by the covariates.

The individual level variables were categorical and median micro area household income was continuous. To simplify equation 2, categorical covariates are represented as a single variable, but the full equation contained variables for each category (excluding the referent group). The age groups were 12-19, 20-29, 30-39, 40-49, 50-59, 60-69, 70-79, 80 and older with the 50-59 year old age group (i=5) as the referent group. Similarly, the CCHS survey cycle categories were cycles 1.1, 2.1, 3.1, 2007-2008, and 2009-2010 with cycle 1.1 (j=1) as the referent group. Micro area median household income (x_k) was mean-centered.

In Bayesian inference, specification of the prior distributions is required to commence modeling. Uniform priors were specified for the random effects, as per equations 3 and 4:

$$u_k|u_{l\neq k}\sim N(\sum\nolimits_{l\in\varphi_k} u_l/n_k \ \tau_a^2) \tag{3}$$

$$v_k\sim N(0,\tau_b^2) \tag{4}$$

where u_k (k=1,…,1,026) models the micro area level spatially correlated random effect for micro area k having l neighbours, a set of immediately adjacent neighbours φ_k , n_k number of

neighbours for micro area k, and variance τ_a^2, with u_k constrained to sum to zero. This specification is based on the conditional autoregressive (CAR) approach, which pools information from immediately adjacent micro areas, and thus has a smoothing effect, and is robust [3]. The uncorrelated random effect, v_k, was specified as Normally distributed with a standardized mean of zero and variance τ_b^2. This unstructured random effect allows each micro area to vary independently of its adjacent neighbours. The variances (τ_a^2, τ_b^2) were specified in another level of the model with uniform distributions having a range between 0 and 100.

The model parameters were derived from three chains of Markov chain Monte Carlo (MCMC) simulations. The first 500,000 simulations in each chain were discarded (burn-in) and the next 50,000 samples were kept. Every 10th sample was saved to estimate the model parameters, for a total of 5,000 saved iterations. Chain convergence was assessed using traceplots, autocorrelation plots, and the Gelman-Rubin statistic [4].

Model Prediction

To derive a neighbourhood-based population-scaled risk factor prevalence estimate, the individual-level modeled probabilities for the 8 age groups (β_i), 5 survey cycles (γ_j), median micro area household income (x_k) and random effects (u_k, v_k) were obtained for all possible combinations (total combinations = 8 age groups x 5 cycles = 40). These individual-level probabilities were obtained for each MCMC sample in each DA in the study area using the following equation:

$$p_{ijk}^{mn} = \frac{\exp(\alpha^{mn} + \beta_i^{mn} + \gamma_j^{mn} + \delta^{mn} x_k^{mn} + u_k^{mn} + v_k^{mn})}{1 + \exp(\alpha^{mn} + \beta_i^{mn} + \gamma_j^{mn} + \delta^{mn} x_k^{mn} + u_k^{mn} + v_k^{mn})} \quad (5)$$

where p_{ijk}^{mn} is the predicted risk factor probability in an individual of age group *i*, survey cycle *j* residing in micro area *k* for each MCMC chain *m* and sample *n*. The variables are otherwise as

defined in equation 2. These probability estimates for each saved MCMC chain and sample were then scaled by CCHS survey cycle and Census populations for a multi-level prediction model via the application of post-stratification methods [5], which are described below. Any micro areas that were not part of the CCHS sampling frame (i.e. Aboriginal Reserves), had missing risk factor data, or had suppressed census data were excluded (N=1,111–1,026=85 micro areas for current smoking in model 2 for males).

The five CCHS cycles were scaled equally at 1/5 or 0.20. Age group scales were the proportion of the neighbourhood population within each age category based on the 2006 Census population. For example, if 10% of the population was in the 40-49 year old age group for a given micro area, a scale of 0.10 was used. The cycle and age group scales were applied across each combination of individual-level probability estimate (40) within each micro area (1,026), MCMC chain (3) and sample (5,000), summed and then divided by the micro area population to derive micro area prevalence estimates for each MCMC chain and sample using the following equation:

$$prev_k^{mn} = \frac{\sum_i \sum_j (p_{ijk}^{mn} \times Pop_{ik} \times \frac{1}{5})}{Pop_k} \qquad (6)$$

where $prev_k^{mn}$ is a micro area level MCMC sample risk factor prevalence. Subscripts and superscripts i, j, k, m, n represent age group, survey cycle, micro area (DA) and MCMC chain and sample, respectively. Pop_{ik} is the population within age group i in micro area k, Pop_k is the total population in micro area k, and $\frac{1}{5}$ is the survey cycle. Together, these factors scale the prevalence estimates to a) reflect that individuals from various age groups do not have equal probability of being sampled in the survey and b) treat each survey cycle equally since they are independent samples.

Finally, the posterior mean of each of these micro area level risk factor prevalence MCMC sample estimates was calculated to obtain the population- and cycle-scaled estimate:

$$prev_k = \frac{\sum_{mn} prev_k^{mn}}{N_k^{mn}} \tag{7}$$

where $prev_k$ is the micro area level posterior mean estimate of the risk factor prevalence, the subscripts and superscripts are defined as before: $prev_k^{mn}$is from equation 6 and N_k^{mn} is the total number of MCMC samples by micro area (15,000). Each estimate ($prev_k$) is the smoothed, predicted micro area prevalence of a risk factor based upon individual response variables in the CCHS, median micro area household income, and the spatially correlated and uncorrelated random effects, scaled by survey cycle and population.

The Bayesian approach can be influenced by the user-specified priors when there is insufficient data to inform the posterior distribution. To verify that the results were not sensitive to the choice of priors, the models were re-run using priors based on the Gamma distribution (shape=0.5, rate=0.005). Comparing the results from different prior specifications, the micro area posterior estimates had low root mean square deviations (all <0.07). Additionally, sample plots for model 2 demonstrate that the micro area prevalence estimates were similar for each prior, as shown in the Figure.

Figure: Density Plots of Risk Factor Posterior Estimates for Gamma vs. Uniform Hyperpriors

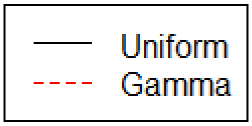

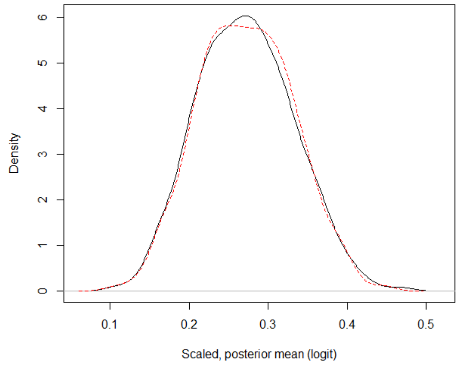

Current smoking, males

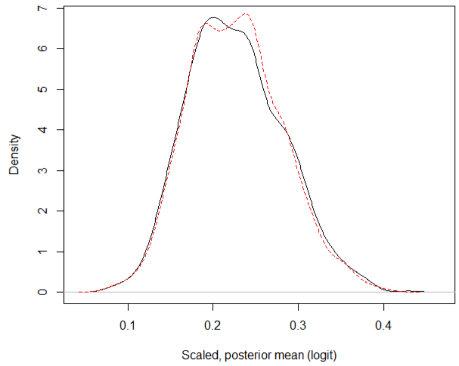

Current smoking, females

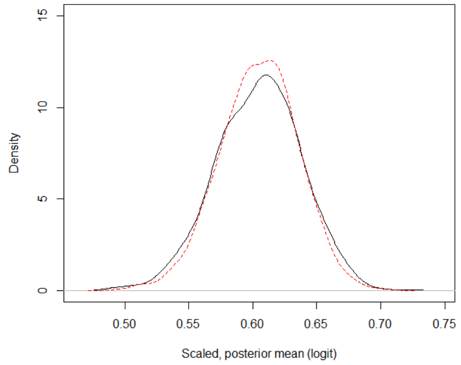

Excess bodyweight, males

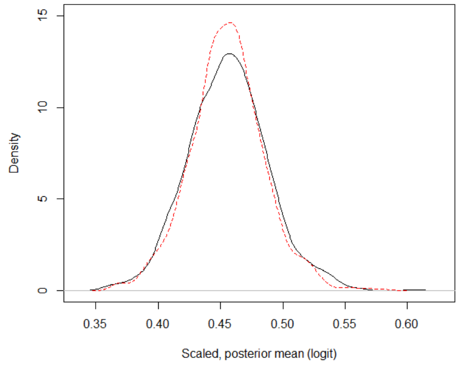

Excess bodyweight, females

References

1. Besag J, York J, Mollie A: Bayesian image restoration, with two applications in spatial statistics. Ann Inst Statist Math 1991, 43:1-59.

2. Statistics Canada: 2006 Census of population. Ottawa: Statistics Canada, 2006.

3. Lawson AB: Bayesian Disease Mapping: Boca Raton: Chapman & Hall; 2009.

4. Brooks SP, Gelman A: General methods for monitoring convergence of iterative simulations. J Comput Graph Stat 1997, 7(4):434-455.

5. Zhang X, Holt JB, Lu H, Wheaton AG, Ford ES, Greenlund KJ, Croft JB: Multilevel regression and poststratification for small-area estimation of population health outcomes: a case study of chronic obstructive pulmonary disease prevalence using the behavioral risk factor surveillance system. Am J Epidemiol 2014, 179(8):1025-1033.
