# Supplementary material for: Estimating micro area behavioural risk factor prevalence from large population-based surveys: a full Bayesian approach
Source: BMC Public Health. 2016 Jun 7;16:478. doi: 10.1186/s12889-016-3144-4 (PMC4897930; doi:10.1186/s12889-016-3144-4)
Supplement: Additional file 2: — Erie-St. Clair Region (PDF 192 kb) [file 12889_2016_3144_MOESM2_ESM.pdf]

Additional File 2: Erie-St. Clair Region

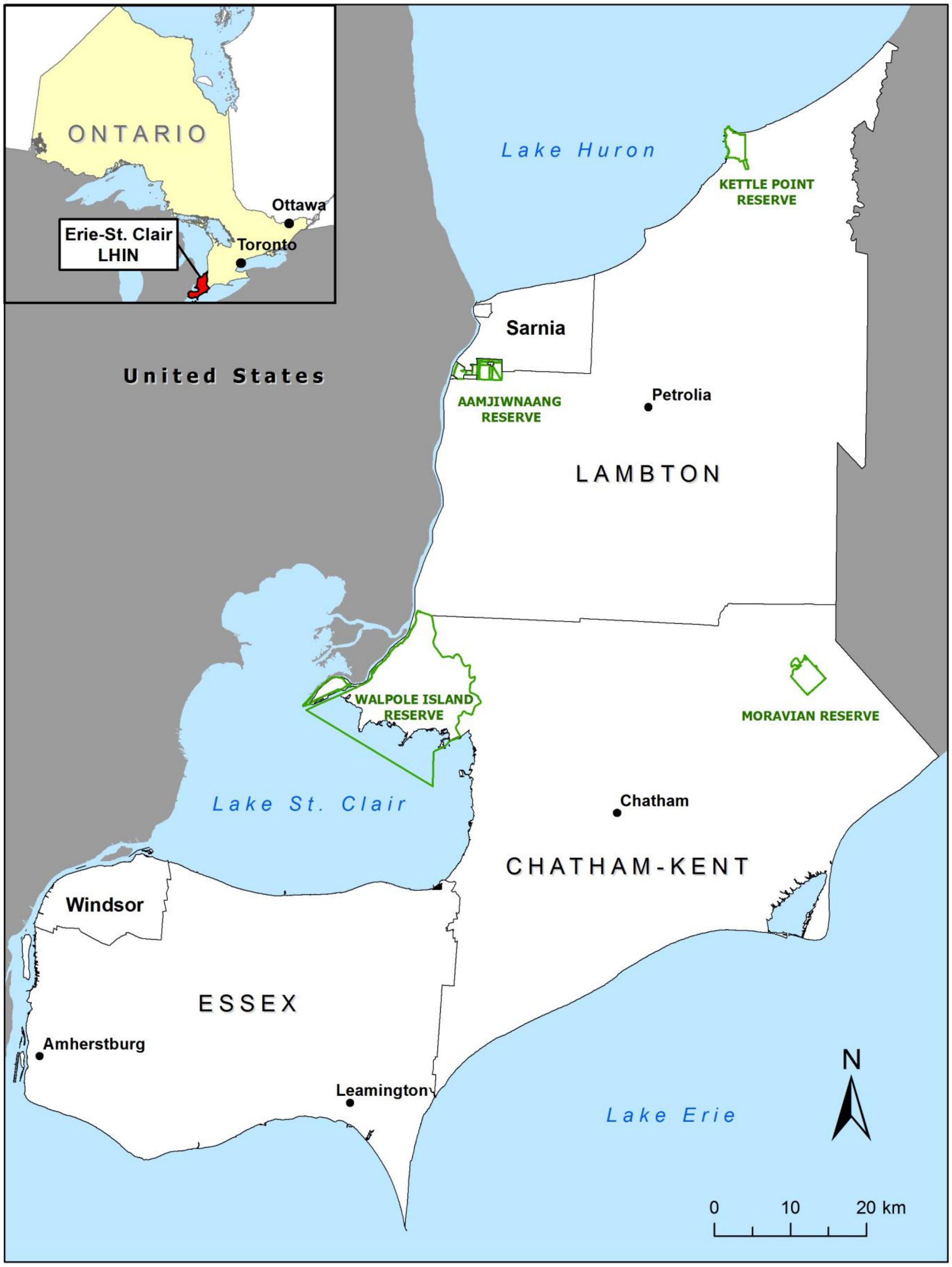

The study area is located in southwestern Ontario, and is comprised of 3 counties which align with the public health units: Lambton, Chatham-Kent and Essex. The region has four aboriginal reserves: Kettle Point, Aamjiwnaang, Walpole Island and Moravian, outlined in green. In 2006, Erie-St. Clair had a population of nearly 630,000 people [1]. Sarnia and Windsor are the largest cities with 56.7% of the population in the region [2]. A large portion of the population lives in urban areas (79.8%), with a minimum population of 1,000 people and a density of at least 400 people per square kilometre [3]. The remaining (20.2%) of the population lived in rural areas

Sources:

1. Statistics Canada. *Age and Sex for the Population of Canada, Provinces, Territories, Census Divisions, Census Subdivisions, and Dissemination Areas*. 2006 Census. Beyond 20/20.
2. Statistics Canada. 2007. *Population and Dwelling Counts, for Canada, Provinces and Territories, and Urban areas, 2006 and 2001 Censuses - 100% Data (table).* Statistics Canada Catalogue no. 97-550-XWE2006002. Ottawa.
3. Statistics Canada. *2006 Census Dictionary.* Catalogue no.92-566-X. Ottawa, Minister of Industry; 2010.
