# Supplementary material for: Estimating micro area behavioural risk factor prevalence from large population-based surveys: a full Bayesian approach
Source: BMC Public Health. 2016 Jun 7;16:478. doi: 10.1186/s12889-016-3144-4 (PMC4897930; doi:10.1186/s12889-016-3144-4)

Median Household Income* from the 2006 Census for the Erie-St. Clair LHIN by 2006 Dissemination Area (N=1,111)

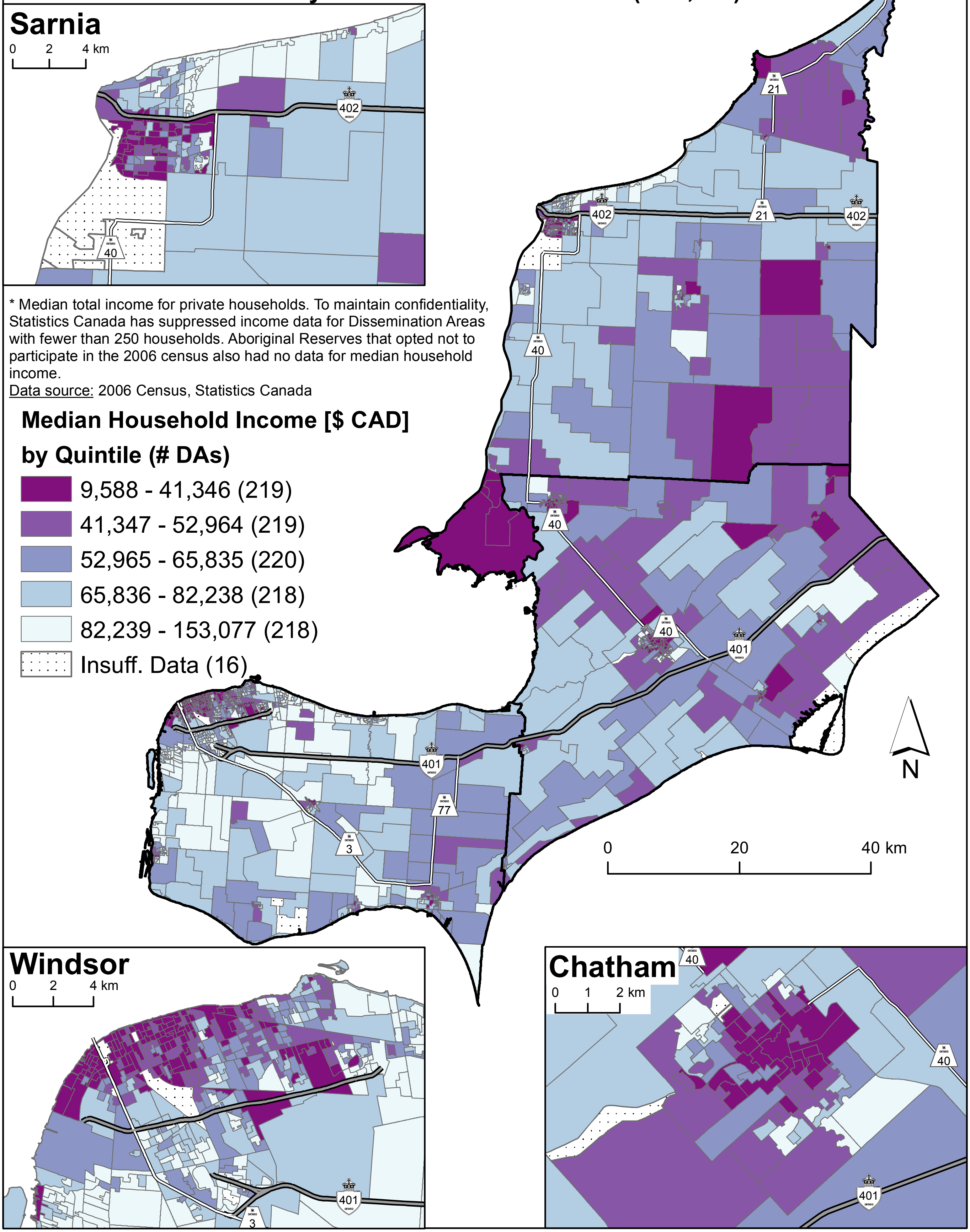

Supplement: Additional file 3: — Median Household Income from the 2006 Census for the Erie St. Clair LHIN, by 2006 Dissemination Area(PDF 1788 kb) [file 12889_2016_3144_MOESM3_ESM.pdf]
