# Supplementary material for: Estimating micro area behavioural risk factor prevalence from large population-based surveys: a full Bayesian approach
Source: BMC Public Health. 2016 Jun 7;16:478. doi: 10.1186/s12889-016-3144-4 (PMC4897930; doi:10.1186/s12889-016-3144-4)
Supplement: Additional file 4: — Spatial Clustering of Behavioural Risk Factors for CCHS Respondents in the Erie-St. Clair Region (PDF 30 kb) [file 12889_2016_3144_MOESM4_ESM.pdf]

Additional File 4: Spatial Clustering of Behavioural Risk Factors for CCHS Respondents in the Erie-St. Clair Region

Behavioural Risk Factor	Observed Number from CCHS	Expected Number	Observed/ Expected	Relative Risk	p-value	Number of DAs	2006 DA population aged ≥ 12 years	Mean Prevalence Within Cluster: Model 1 (%)	Mean Prevalence Within Cluster: Model 2 (%)
**Current Smoking**									
Males									
Sarnia	147	85.6	1.70	1.76	<0.001	39	8 195	37.7	35.7
Windsor	152	97.5	1.56	1.61	<0.001	87	18 617	34.5	34.0
Chatham	106	65.0	1.67	1.67	0.002	38	8 852	32.7	32.8
Females									
Sarnia	188	123.9	1.52	1.58	<0.001	64	15 082	28.1	27.8
Windsor	188	129.0	1.46	1.51	0.001	118	25 557	27.6	29.2
**Excess Bodyweight**									
Males									
Windsor	453	389.8	1.16	1.18	0.01	129	34 965	64.4	64.6

Notes: The observed number within a cluster was the sum of CCHS respondents with the behavioural risk factor. The expected number within the cluster was determined from the Bernoulli likelihood function for binary outcomes under the null hypothesis of no variation in the risk factor prevalence inside and outside the cluster [54]. SaTScan provided a relative risk estimate, which was the ratio of the observed/expected value within the cluster divided by the observed/expected value for the area outside the cluster. DA-level population estimates were obtained from the 5-year age groups provided by the 2006 Census. Sixty percent of the 10-14 year old age group was used to obtain population counts for 12-15 year age group to correspond to the age groups included in the CCHS (≥12 years). The mean modeled prevalence estimates were obtained from the post-stratified estimates for DAs within each cluster for models 1 and 2.

Abbreviations: CCHS = Canadian Community Health Survey; DA = Dissemination Area (from 2006 census);
