# Supplementary material for: Estimating micro area behavioural risk factor prevalence from large population-based surveys: a full Bayesian approach
Source: BMC Public Health. 2016 Jun 7;16:478. doi: 10.1186/s12889-016-3144-4 (PMC4897930; doi:10.1186/s12889-016-3144-4)
Supplement: Additional file 5: — Mean Micro Level Behavioural Risk Factor Prevalence Estimates and 95 % Credible Intervals for the Erie-St. Clair Region (PDF 143 kb) [file 12889_2016_3144_MOESM5_ESM.pdf]

## Additional File 5: Mean Micro Level Behavioural Risk Factor Prevalence Estimates and 95% Credible Intervals for the Erie-St. Clair Region

### Current Smoking Prevalence

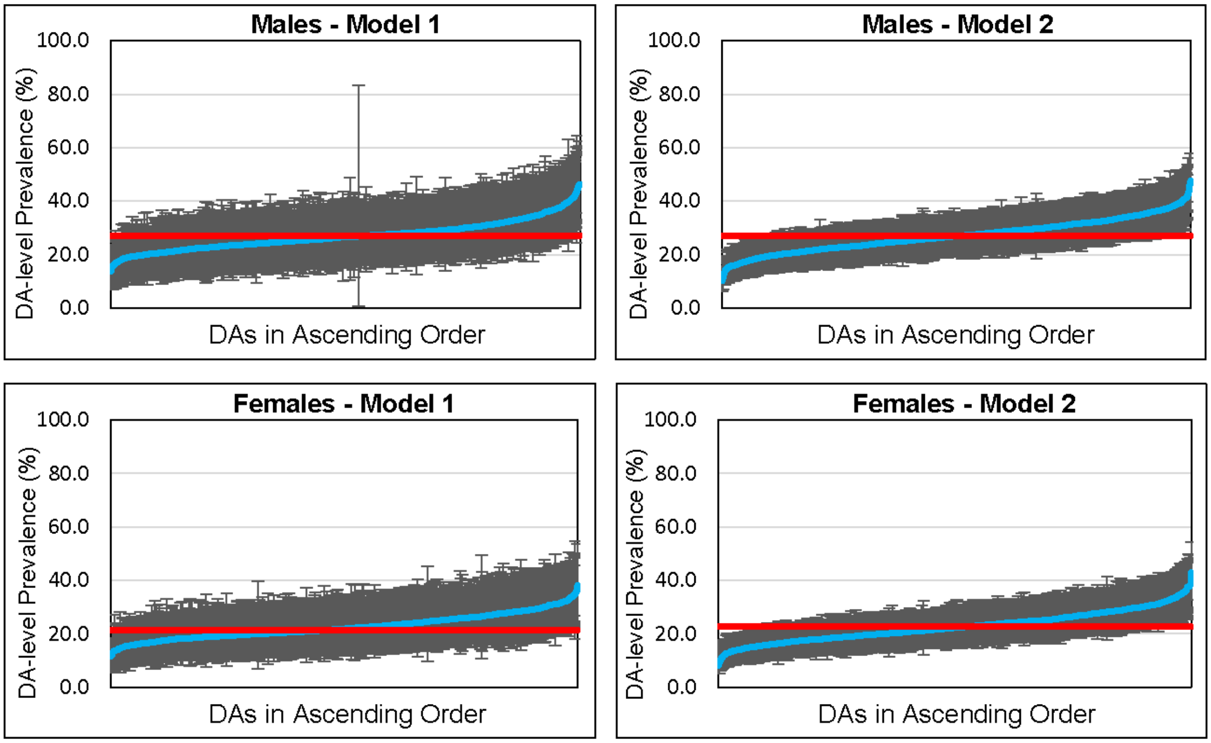


Post-stratified modeled current smoking prevalence estimates from the Bayesian posterior samples are shown, with the Dissemination Areas (DAs) in ascending order, from lowest to highest modeled prevalence. For each DA, the mean prevalence of the posterior distribution is shown in blue, and the 95% credible intervals (2.5$^{th}$ and 97.5$^{th}$ percentiles) are shown in grey, with results for males on the top, and females on the bottom. For model 1 (left), the red line represents the model-based mean current smoking prevalence for Erie-St. Clair (25.7% for males, 21.3% for females). For model 2 (right), the red line represents the mean model-based current smoking prevalence for Erie-St Clair (27.1% for males, 22.6% for females).

## Excess Bodyweight Prevalence

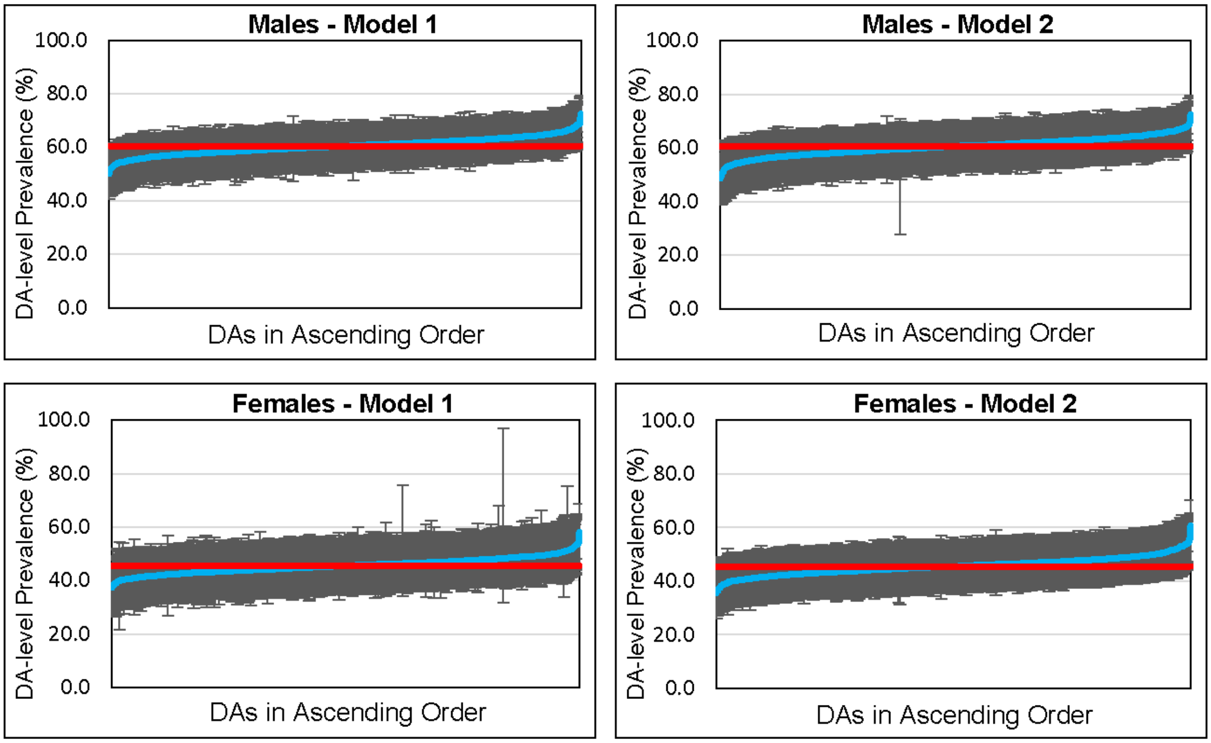


Post-stratified modeled excess bodyweight prevalence estimates from the Bayesian posterior samples are shown, with the Dissemination areas (DAs) in ascending order, from lowest to highest modeled prevalence. For each DA, the mean prevalence of the posterior distribution is shown in blue, and the 95% credible intervals (2.5$^{th}$ and 97.5$^{th}$ percentiles) are shown in grey, with results for males on the top, and females on the bottom. For model 1 (left), the red line represents the model-based mean excess bodyweight prevalence for Erie-St. Clair (model-based: 60.5% for males, 45.6 % for females). For model 2 (right), the red line represents the model-based mean excess bodyweight prevalence for Erie-St. Clair (60.5% for males, 45.6% for females).
